# The role of MRI biomarkers in evaluation of symptomatic pineal cysts – a retrospective analysis

**DOI:** 10.1007/s00701-024-06212-w

**Published:** 2024-08-03

**Authors:** S. Greisert, S. Fleck, E. Rathmann, M. Vollmer, H. W. S. Schroeder

**Affiliations:** 1 Neurosurgical Department, University Medicine Greifswald, Greifswald, Germany; 2 Radiological Department, University Medicine Greifswald, Greifswald, Germany; 3 Institute of Bioinformatics, University Medicine Greifswald, Greifswald, Germany

**Keywords:** Pineal cyst, Cerebral edema, Intracranial hypertension, Magnetic resonance imaging, Apparent diffusion coefficient

## Abstract

**Background:**

Our aim was to determine whether the Apparent Diffusion Coefficient is able to predict the presence of a symptomatic pineal cyst by detecting cerebral edema.

**Methods:**

We retrospectively analyzed MRIs of 45 patients with pineal cysts before and after resection and 51 patients without pineal cysts, comparing ADC values of thalamus, central, periventricular and subcortical white matter. Furthermore we evaluated cyst size and morphology and analyzed its correlation to ADC values in corresponding patients.

**Results:**

Differences between patients with symptomatic pineal cyst and control group were not significant (*p* = 0.200 – 0.968). ADC ratios did not change significantly after resection of the cyst (*p* = 0.575 – 0.862). Cyst size showed no significant correlation to ADC ratios (*p* = 0.071 – 0.918). Raw data analyses revealed more significance, especially periventricularly and in central white matter, which resulted in significant interhemispheric differences in ADC ratios in both subgroups (*p* < 0.001 and *p* = 0.031). MRI of 1.5T showed consistently higher values than 3T but mostly insignificant.

**Conclusion:**

Our analysis revealed no evidence that pineal cysts lead to intracerebral edema caused by venous compression. Since variability was higher than the differences seen, ADC sequences do not appear to be an appropriate diagnostic tool for symptomatic pineal cysts.

## Introduction

In a series of studies Eide and Ringstad proposed that ADC values could be used as MRI biomarkers to detect intracranial hypertension due to venous congestion as the reason for symptoms arising from pineal cysts in the absence of hydrocephalus [4–6]. The authors suspected that pineal cysts, becoming clinically apparent especially in the form of headache, visual disturbance, nausea and vomiting, might be a hindrance for venous blood drain from central cerebral areas caused by compression of the internal cerebral veins. Thus they tried to detect differences in cerebral diffusion of patients with pineal cysts and chronic headache patients without cysts [5] quantifying edema by the Apparent Diffusion Coefficient [4]. Thalamus, periventricular and subcortical white matter were investigated and set in relation to central white matter to form an ADC ratio for minimizing bias due to technical differences in MRI implementation. Eide and Ringstad found a significant correlation between symptom severity and severity of proposed venous hypertension measured and graded by ADC ratios in combination with the TSCR as a marker for crowding within the pineal recess [4]. A comparison of PC patients with a control group of patients suffering from unclear chronic headache consisted of about 20 patients only per group and showed slightly different results [5].

The analysis of diffusion weighted MRI such as DWI and ADC has become an established tool in the diagnostics of venous thrombosis [11], stroke [1] or idiopathic intracranial hypertension [14], but has also been looked at as a possible biomarker for other pathologies such as normal pressure hydrocephalus or degenerative diseases [10, 12]. Emphasis has been laid on diffusion weighting these last years for that it is able to discriminate between different types of edema originating from diverse pathologies within the brain making it an additional radiological marker for diagnosis and prognosis [13].

Symptomatic pineal cysts in the absence of hydrocephalus are still a rather clinical than radiological diagnosis. Indication for surgical resection is very difficult due to variable and unspecific symptoms reported by the patients. A specific radiological marker would be very helpful in objectifying this often very uncertain diagnosis.

## Methods

### Study design

In a retrospective analysis we tried to detect differences in cerebral diffusion measured by diffusion weighted MRI sequences as an indicator for deficits in venous drainage between patients with and without symptomatic pineal cysts. Our hypothesis was that patients with symptomatic pineal cysts show higher ADC values—especially thalamic and periventricular—than patients without pineal cysts and thus underline the findings of Eide and Ringstad [5]. Our goal was to determine whether ADC ratios as formed by our Scandinavian colleagues could function as an objective diagnostic tool for detecting a symptomatic Pineal cyst. Furthermore we wanted to figure out if there is covariates – of technical or epidemiological nature – that would possibly bias the results. Therefore we compared patients with and without symptomatic pineal cysts, pineal cyst patients pre- and postoperatively, try to reproduce the correlation of cyst size and ADC values and do a raw data multivariate analysis.

### Patients

From our databank which was raised retrospectively before 2016 and prospectively thereafter we extracted 79 patients with histopathologicaly assured diagnosis of pineal cyst with and without hydrocephalus of all age, both male and female operated from October 2003 to January 2022. The diagnosis of symptomatic pineal cyst was ensured by clinical improvement after resection in all patients included in our analysis. 74 of the patients were operated via suboccipital craniotomy and infratentorial-supracerebellar approach (3 subtotally and 71 totally) [2, 8]. Only 45 of those patients had MRI with ADC sequence available and were included in this study. Indication for resection was always provided by the last author (HS) generally based on strong severity of symptoms, high pressure of suffering and long patient history of frustrating conservative therapy attempt. Surgery was in almost all cases done by the last author (HS), in some occasions by the second (SF), severe complications did not occur in any of the cases included.

As control we used patients from our department who were hospitalized and went through radiological diagnostics for other reasons than pineal cysts, including trigeminal neuralgia, hemifacial spasm, meningiomas, vestibular schwannomas, craniopharyngiomas, pituitary adenomas, light cerebral contusion, arachnoid cysts or were under follow up postoperatively after surgical therapy due to one of the mentioned. Patients were randomly chosen, but we tried to match for age and gender knowing that most of our patients with pineal cyst are of young mature age and female. Care was taken that the pathologies did not affect our regions of interest and that there was no contact to the venous system and no disturbances of CSF circulation. Those patients will be referred to as “controls “ or “others “.

### MRI and data collection

For collection of data DeepUnity Viewer (Dedalus Healthcare GmbH, Bonn, Germany) was used as radiological software program for image analysis. Field strength and slice thickness were extracted for subgroup analyses. We measured the apparent diffusion coefficient and cyst size using the latest MRI with ADC sequence available before resection as well as the last available MRI in follow up. Values are given in 10^–6^ mm^2^/s.

Data extracted included facts regarding the technical implementation like manufacturer and model of the device used, field strength and slice thickness. By subgroup analysis we tried to figure out if there are differences arising.

Cyst size was measured in all three planes. While width was measured as the highest width in axial plane, height and length were measured in midsagittal plane. Tectum-splenium/cyst ratio (TSCR) was measured in midsagittal plane at the point of highest narrowing and is given in percents of space between tectum and splenium occupied by the cyst as described by Eide and Ringstad [5]. T2-sequences were used for evaluation of cyst morphology.

**Fig. 1:**
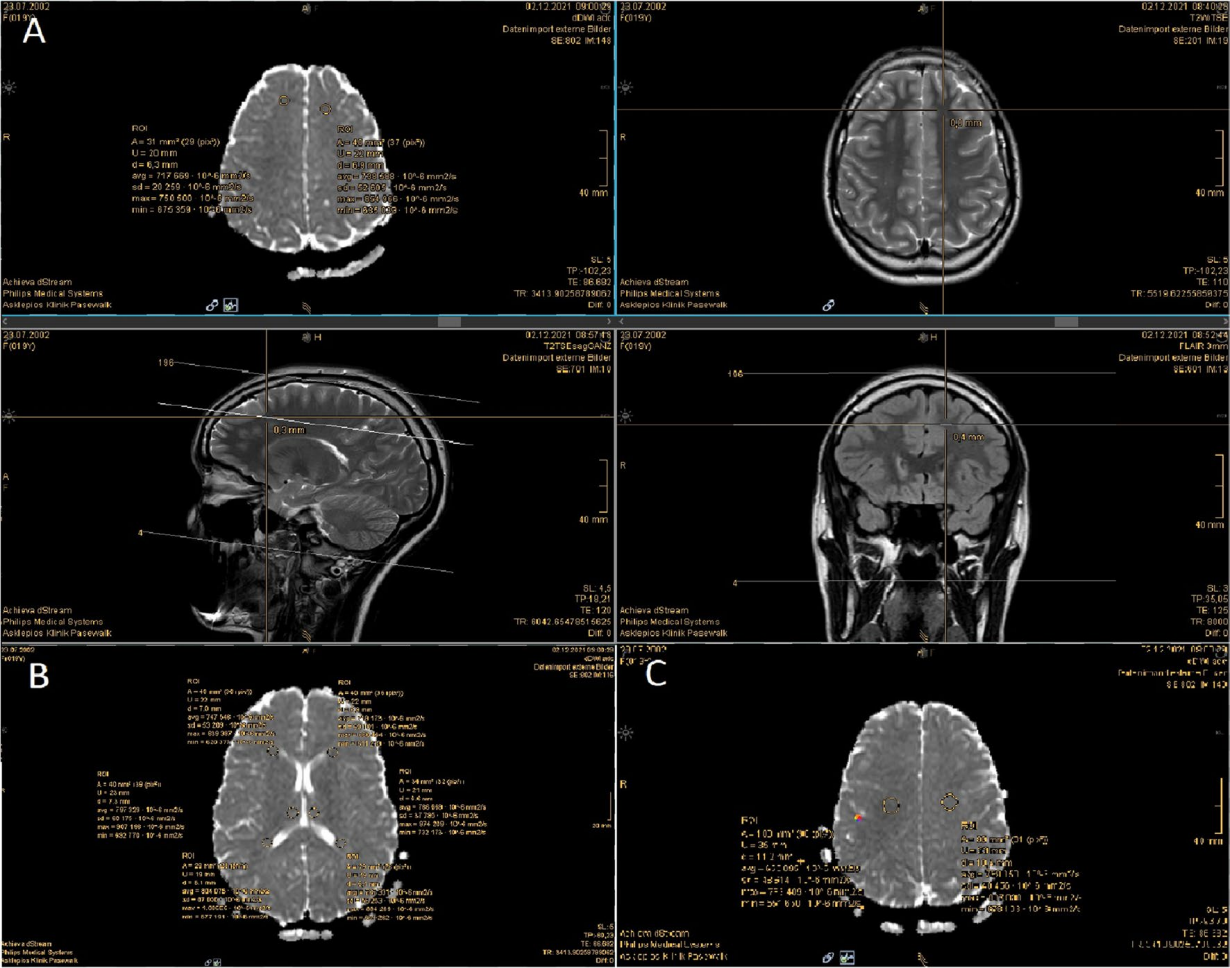
A: region of interest was determined in axial ADC maps being combined with T2 images in all planes using the “Livesync”-function. In this example subcortical ROIs were set. B: positioning of thalamic and periventricular ROIs. C: setting of ROI in white matter

Measures of ADC values were taken under supervision of our neuroradiologist and in accordance with Eide and Ringstad [4]. All ROIs were set in both hemispheres in axial plane of an ADC map, using the “Livesync”-function with T2 images when available in axial, sagittal and coronal plane to make sure the correct ROI alone was selected. Here thorough care was taken to avoid neighboring tissue of cortex, basal ganglia and CSF system, especially partial volume effects were thoroughly avoided. Therefore periventricular and subcortical ROIs varied slightly (± 5mm periventricularly and ± 10mm subcortically) in anatomical position in some patients. Thalamic ADC values were taken in the most cranial cut part of the thalamus where it was still precisely distinguishable from adjacent choroid plexus of the lateral ventricles. Periventricular ADC values were taken at the same level or 3mm above, depending on where parenchyma appeared clearer distinguishable from partial volume of the CSF system. ROIs were set at both the frontal and occipital horns of the lateral ventricles. Central white matter was measured 3mm above the most cranial slice showing parts of the side ventricles and subcortical measures 3-6mm above in frontal white matter next to the cortex. (Fig. 1) All ADC ratios were calculated using white matter as reference to the others stated above and determined on each side separately. If not differently stated values represent the mean of both hemispheres. Periventricular ratios refer to the mean of all four values. Other than Eide and Ringstad who proposed a cut off ratio of > 1.0 for detection of cerebral edema [4] we set our cut off > 1.1.

Eide and Ringstad analyzed ratios in reference to white matter instead of raw data, proposing that bias might arise from comparing MRI with different field strength. We therefore performed another subset of analyses including MRI with a field strength of 1.5 Tesla only comparing raw data of ADC values from the previously stated.

### Clinical data

Only the presence or freedom of headache in both patient groups with and without pineal cysts was included as clinical data for subgroup analysis. As stated before for our cyst positive cohort postoperative improvement of headache and other symptoms was the inclusion criterion.

### Statistics

We used STATA 13 (StataCorp LLC, College Station, Texas, USA) for statistical analyses of data and all graphical depictions. For analyzing continuous data and for multivariable analyses we used linear regression and multifactorial ANOVA. For two-sample-comparisons we used paired and unpaired t-tests and for categorial data analyses we used the exact Fisher test. Significance level was set to alpha < 0.05.

## Results

### Power estimation

Prior to analysis we did a power estimation based on the findings of Eide and Ringstad [5]. Comparing mean and 95% CI for the region of least effect size and statistical significance from both the pineal cyst and the chronic daily headache cohort we calculated a number of 41 patients per group at least to achieve a power of 80% and significance level of 95%.

### Summary

A total of 45 PC patients with preoperative imaging including ADC sequence were included, of which 2 were not included in postoperative analysis due to missing ADC senquences. The control group of patients without symptomatic pineal cyst included 51 patients. Mean age of the PC group was 27.9 years compared to 29.6 years in the control group. 82% of our patients with PC were female, among controls they were 71%. 44 of our 45 PC-patients (98%) suffered from headache while only 19 of the 51 (37%) controls did so according to history at presentation, leading to MR imaging. Signs of hydrocephalus were seen in three patients with pineal cyst (7%). Other symptoms found in PC patients included sleeping disorders (42%), visual disturbances (53%), intermitting nausea and vomiting (73%) as well as dizziness (53%). 36 patients showed significant relief of symptoms postoperatively, 9 showed some relief (20%).

Among our control group four patients showed an incidental finding of pineal cyst ranging from 9.8 to 14.9mm max diameter, mean TSCR 69% (57 – 79%). Only one reported acute headache due to light cerebral contusion.

### Comparison of ADC ratios among symptomatic PC patients and controls

**Fig. 2:**
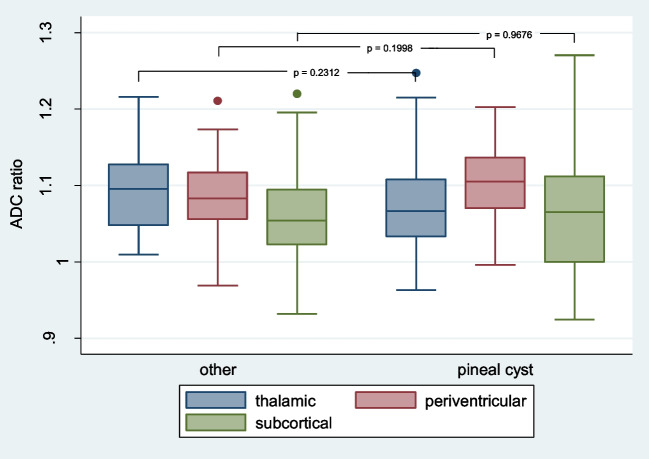
Depicted are the median and values within 25th and 75th percentile for thalamic, periventricular and subcortical ADC ratios in PC-patients and others, adjacent significance levels after univariate ANOVA

Mean thalamic, periventricular and subcortical ADC ratios were 1.079, 1.1 and 1.06 respectively in patients with PC. In others they were not significantly different with 1.093, 1.087 and 1.061 ( *p* = 0.200 – 0.968). Also minimum and maximum values did not vary significantly. The lowest ratio observed was 0.925 subcortically in one PC patient. Both groups showed maximum ADC ratios in all regions of interest of more than 1.2 (highest observed was 1.27 in PC-patient). (Fig. 2) Unihemispherical analysis of the marked ROIs showed differences.

Using Fisher´s exact test we tried to figure out whether patients with symptomatic pineal cyst reached an ADC ratio of > 1.1 more frequently than others. In thalamic and subcortical ADC ratios no significance was found (*p* = 0.302 and *p* = 0.482). Analysis of periventricular ADC ratios > 1.1 showed a statistical significance (*p* = 0.023) with 57% of PC patients having ADC ratios > 1.1 and only 33% in others. This significance could not be reproduced in unihemispherical analysis of frontal and occipital periventricular ratios (*p* = 0.189 and *p* = 0.101), however PC patients more frequently had ratios > 1.1 (40% and 57% compared to 25% and 39% respectively).

To figure out the possible influence of covariates we used multifactorial ANOVA including presence of headache, gender and age. In this case presence of pineal cyst showed little significant influence on subcortical ADC ratios (*p* = 0.040), but no significant difference for all other regions (*p* = 0.055 – 0.958).

To make sure that the coincidental finding of a pineal cyst in 4 patients of our control group did not bias our results we repeated our analysis excluding those patients. Results and *p*-values remained about the same (*p* = 0.248, *p* = 0.320 and *p* = 0.809 for thalamic, periventricular and subcortical ratios respectively).

### Comparison pre- and post-interventional in PC patients

**Fig. 3:**
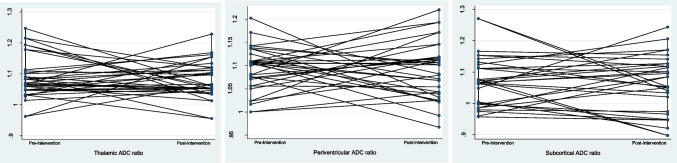
Pre- to postoperative change of thalamic, periventricular and subcortical ADC ratios. Radiological follow-up was 66 to 1210 days, no patient showed a recurrent pineal cyst. (from left to right: *p* = 0.856, *p* = 0.575, *p* = 0.862)

Average ADC ratios among patients with PC did not change after resection of the cyst. Mean difference was insignificant for thalamic, periventricular and subcortical ADC ratios bihemispherically as well as unihemispherically (p = 0.323 – 0.972). The average time to postoperative follow-up was 120.4 days. 21 patients only had postoperative follow-up from day 1 – 4 available. A subgroup analysis on the remaining 22 patients with 2 months and more till follow-up, accounting for possible postoperative effects like swelling and edema as a reason for bias, still showed no significance (*p* = 0.252 – 0.915). No recurrence of pineal cyst was observed in any of the patients. Apparent diffusion coefficients seemed to change randomly into one or the other direction after the operation. (Fig. 3).

Furthermore we did not see ADC ratios of > 1.1 becoming less frequent after resection. In all cases of thalamic, periventricular and subcortical ADC ratios, we found frequency even rising but insignificantly (*p* = 0.519—0.830).

In a total of 10 patients field strength of conducted MRI differed from pre- to postoperatively. While seven patients received preoperative MRI of 3T, three other individuals did so postoperatively. All others were done using 1.5T.

### Correlation of cyst size and ADC ratios

Mean width of the cysts was 12.9mm (7-24mm), mean of maximum length was 16.7mm (6-27mm), mean of maximum height was 12.3mm (6-21mm). Mean TSCR among patients with PC was 87% (64 – 100%). The occupation of space between tectum and splenium by the pineal gland with and without cyst among our control group patients was 48% (20 – 79%), excluding those with incidental findings of a cyst the mean decreased to 46% (20–74%).

**Fig. 4:**
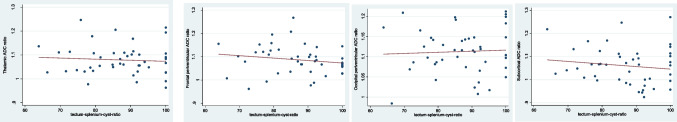
Correlation of TSCR and ADC ratios for PC patients only. No statistical difference was seen (from left to right: *p* = 0.628, *p* = 0.265, *p* = 0.746, *p* = 0.367)

Analyzing PC patients only, TSCR correlated inversely with the thalamic, frontal periventricular and subcortical ADC ratios. Positive correlation was found between TSCR and occipital periventricular ADC ratios. Therefore in this analysis periventricular ADC ratios from frontal and occipital area were analyzed separately. All of them were statistically not significant (*p* = 0.265 – 0.746). (Fig. 4).

**Fig. 5:**
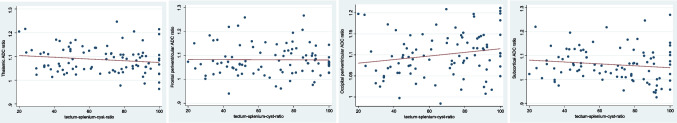
Correlation of TSCR and ADC ratios for both PC patients and controls. No statistical difference was seen (from left to right: *p* = 0.160, *p* = 0.918, *p* = 0.071, *p* = 0.208)

Other than Eide and Ringstad who by default set all control group patients to a TSCR of 0, we extracted the size of the pineal gland and space between tectum and splenium and calculated the ratio for our analysis. An analysis on correlation of TSCR and ADC ratios including both PC patients and others lead to similar results. Positive correlation between occipital periventricular ADC ratio and TSCR was seen, not reaching statistical significance (p = 0.071). All other ROIs showed negative correlation again (*p* = 0.160 – 0.918). (Fig. 5).

The pineal region is anatomically very variable. Tectum-splenium-distance varied from 5.9 – 18.1 mm (mean 8.9 mm) in the control group. For validation analysis was repeated with maximum extension of the cystic formation in all dimensions instead of ratio in PC patients. Results showed negative correlation mostly and no significance. (*p* = 0.085 – 0.946).

### Raw data analysis and additional findings

In our raw data analyses we compared individuals having been examined by MRI with a field strength of 1.5 Tesla only, leaving 38 patients with pineal cyst and 48 control group patients.

### PC patients and controls

Periventricular ADC values from all 4 ROIs were higher in PC patients than in the control group. Differences reached statistical significance for left frontal periventricular ADC values (*p* = 0.037) and right occipital periventricular (*p* < 0.001), slightly missed significance for left occipital periventricular (*p* = 0.056) and showed no significant difference for right frontal periventricular ADC values (*p* = 0.174). Right hemispheric white matter showed significant higher ADC values in PC patients (*p* = 0.017), all others remained insignificant (*p* = 0.167 – 0.776).

### Pre- and postoperative values

Comparison of pre- and postoperative ADC values in PC patients using an unpaired t-test showed a significant reduction of mean ADC value for periventricular frontal ROI on the left (*p* = 0.025), the rest remained insignificant (p = 0.196 – 0.897). ADC values even increased or did not change after surgery in 70% of the analyses made. Only 35 patients were included in the course of subgroup analysis for field strength of 1.5T.

### Correlation of cyst size and ADC values

Analysis of the correlation between Tectum-splenium/cyst ratio and raw ADC data using linear regression in both PC patients and controls showed rising ADC values with increasing occupation of the space between tectum and splenium for all ROIs except right thalamic, being significant both left and right occipital periventricularly (*p* = 0.019 and *p* = 0.001) as well as white matter right hemispherically (*p* = 0.013), all others insignificant (*p* = 0.137 – 0.932). In PC patients alone ADC values correlated negatively with space occupation in all ROIs, significantly right thalamic (*p* = 0.015), the rest insignificantly (*p* = 0.055 – 0.761). Correlation of raw cyst size with ADC values in PC patients showed a negative correlation for all dimensions and ROIs (*p* = 0.006 – 0.802), except for right hemispheric white matter which showed insignificantly higher ADC values with increasing length and width (p = 0.824 and *p* = 0.609) but insignificantly negative correlation with height (*p* = 0.987).

### Left and right hemispheric differences

Surprisingly we found significantly higher right hemispheric ADC ratios compared to left hemisphere in PC patients (*p* = 0.031) and even more significant in controls (*p* < 0.001) when compared according to Eide and Ringstad. We therefore analyzed the patients´ raw data. In summary differences were more significant in the control group than in PC-patients. White matter showed higher ADC values in the left hemisphere than in the right but only significant for controls (*p* = 0.001), insignificant for PC patients (*p* = 0.469). Frontal periventricular ADC values were significantly higher on the right side than on the left side for both PC-patients (*p* = 0.049) and controls (*p* = 0.001). The other ROIs showed insignificant lateralization mostly toward the right hemisphere (*p* = 0.070 – 0.924).

### Difference in MRI of 1.5T and 3T

Most MRI (81% in controls and 75% in PC patients) were done using Siemens devices (Symphony, Aera, Verio, Avanto, Espree, Skyra). Other devices used were Philips (Achieva) and General Electrics (MR750w and MR450w), 14% and 5% in controls and 19% and 6% in PC patients respectively. Most MRI was being done using a field strength of 1.5T, only 16% in PC patients and 6% in controls were being done using 3T. Slice thickness ranged from 3 to 5.5 mm. Linear regression for the influence of field strength on raw ADC values matched for ROIs and patient group revealed averagely higher values for 1.5T MRI in almost all analyses, reaching significance in left frontal periventricular values in PC patients (*p* = 0.049) and right thalamic in controls (*p* = 0.037) only. 3T MRI showed higher ADC values in periventricular ROIs within the control group only, but insignificant. A multivariate analysis on the influence of all three technical aspects combined showed similar results.

## Discussion

While asymptomatic pineal cysts are often a coincidental diagnosis, symptomatic cysts seem to be very rare. Even though 75% of the radiological diagnostics revealing the presence of a pineal cyst are done due to headache [9], more often the headache persists for other reasons like migraine or tension headache which show a much higher prevalence among patients with and without pineal cysts.

In the process of data extraction we realized that especially next to the ventricles setting an ROI is not as intuitive as it might seem. Partial volume effects in direct elongation of the ventral and dorsal horns could be easily confused with small interstitial edema forming around the ventricular system and thus lead to wrong interpretation. Screening the literature on diffusion weighted MRI for the diagnostics of cerebral diseases we found out that other authors used different techniques leading to big differences in ADC values. While Eide and Ringstad described a thorough avoidance of these partial volume effects [4], Goujon measured ADC values in direct elongation of and right next to the ventral and dorsal horns regardless of suggestivity for partial volume effects when analyzing differences in diffusion among patient with normal pressure hydrocephalus and neurodegenerative diseases [10]. In another subanalysis we extracted periventricular ADC values after the protocol of Goujon and compared them to the ones extracted after Eide and Ringstad [4] with significant difference (p = 0.000).

Variability in data extraction is high especially in close proximity to neighboring tissue that can falsely be partially included in the ROI since ADC maps show a comparably low resolution. Tools like the “Live Sync” function in the viewer we used seem to be indispensable for consistency in data extraction. Even using this we found a high intra and inter observer variability. Having been sensitized in the course of data extraction and analysis for the variability of ADC values in just slightly different locations we want to emphasize that differences found in the results of our colleagues and us might arise from different setting of ROIs. In particular we seem to have measured thalamic and occipital periventricular ADC values above the ROIs described by Eide and Ringstad [4]. Looking at their figures and description the angle between axial and sagittal plane seems to have been steeper than in the standardized protocol for imaging that we have used. Pineal recess and Vermis cerebelli are clearly visible in one plane with the ventral horns of the side ventricles at the height of the Foramina of Monro while our orientation is more cranial towards the parietal lobe at same height of the ventral horns. Even though we would expect thalamic edema forming equally over the entire Thalamus and being visible in one region as in the other this is a difference to keep in mind.

As mentioned the cut off we chose to define ADC ratios as elevated was at 1.1 which is different to Eide and Ringstad. Almost all ratios acquired from our control group patients were above 1.0 which did hence not allow for a meaningful comparison. In the publication of our Scandinavian colleagues all control group patients with unspecified headache presented ratios of less than 1.1 [5] which in synopsis lead to our decision for choosing a higher cut off. This decision was encourage by Engelter et al. who described physiologically higher thalamic ADC values than that of white matter in healthy patients analyzing differences in elderly patients [7].

In our analysis only the periventricular region seemed to slightly support the hypothesis of higher ADC ratios as indicator for edema in PC patients with significantly more patients showing ratios of  > 1.1 than in the control group and occasional significance in the raw data analysis. At the same time periventricular ADC ratios were the most inconsistent and most prone to bias in data extraction, since areas of visibly different levels of diffusion lie very closely to each other. This is supported by the fact that periventricular ADC values from our observation differed most from that of other authors.

Another important difference is that we did not form subgroups by categorizing between patients with “non-moderate” and “much-severe” symtoms as Eide and Ringstad did [4]. When indication for resection was provided all patients were considered “much-severe” according to our colleagues scale. Furthermore data from patients being operated before 2016 available for this retrospective analyses was not always specific enough to allow for such a thorough categorization. Also we did not devide Pineal cyst into 4 subgrade based on TSCR > 90% and thalamic ADC ratio > 1.0 since almost all of our patients in both the PC and control group showed ADC ratios of > 1.0 and grading as described by Eide and Ringstad [4] did not seem suitable. Therefore data analysed was mainly metric while our colleagues rather formed categorial variables to analyse correlations. This must be kept in mind when comparing results.

In fact we found a negative correlation of ADC values and TSCR in patients with symptomatic pineal cyst. A reason might be that greater pineal cysts could have been present and probably progressive for longer time and therefore lead to chronification and adaption by bypassing circulation. Negative correlation was also found when including control group patients except for occipital periventricular ADC ratio. Anatomically this would not be explained by cyst size and space occupation when considering venous drainage. Analysis of correlation between ADC ratios with raw cyst size in any dimension instead of tectum-splenium/cyst ratio showed similar results. Only positive but mostly insignificant correlation of raw ADC values in all ROIs and TSCR including both PC-patients and controls only slightly supports the hypothesis of impaired drainage and consecutive edema. Other than Eide and Ringstad who numbered TSCR of all their control group patients as zero, we quantified crowding of the pineal recess by forming a ratio between the space between tectum and splenium and size of the pineal gland.

Another interesting just lately published study of our Scandinavian colleagues, showing decreased blood flow velocity over the internal veins as well as glymphatic enrichment of intrathecally applied contrast agent correlating with increasing crowding of the pineal recess and moreover correlating with increased thalamic ADC ratios, further supports the hypothesis of venous blood flow alteration as a possible reason for symptoms in Pineal cyst patients [3]. This study very much highlighted the complex correlations of CSF flow and brain wide venous drainage especially when being disturbed in close vicinity of the pineal recess. It did not correlate though ADC values and symptom severity directly and could not have been compared to our data.

Only cyst size being > 5mm, or space occupation of the space between tectum and splenium being > 50% were consistently observed among all PC-patients while mean TSCR was < 50% in control group patients. Therefore these might be considered minimum radiological requirements for diagnosis of a symptomatic pineal cyst. Our analysis doesn´t suggest that ADC values might be a promising biomarker in radiological imaging suggesting whether the cyst is symptomatic or just an incidental diagnosis accompanied by headache of different pathogenesis. Especially since variability among subgroups was mostly higher than the differences found between PC-positive and negative subgroups, as well as between pre- and postoperative measurements.

Whether field strength applied in MRI affects ADC values and hence requires the calculation of ADC ratios, as suggested by Eide et al., or rather raw data should be used, is a matter of controversy. In our analysis of the raw data we also found significant differences in white matter suggesting that this ROI might be as susceptible to changes in diffusion as the others. Therefore differences might be missed when forming the ratio of two ROIs that increase equally in a group of patients compared to the other, or lead to falsely high results in expected regions if in fact that of white matter is randomly low. The necessity of forming a ratio for the purpose of compensating for differences in MRI resulting from field strengths therefore seems to be questionable. On the other hand all ROIs other than periventricular showed consistently higher values when MRI was done with 1.5T. This would suggest that field strength might have an impact that could bias data, if completely ignored.

From fMRI studies we know about lateralization in the brain, which can also be found in diffusion imaging. Other than Wilde et al. [15], who found either no difference or higher ADC values over all areas of the right hemisphere compared to the left, statistically significant in thalamus and white matter, our analysis showed higher values left hemispherically within central white matter, which reached significance in our control group. Reason might be a difference in data collection. In all other regions our findings were quite similar, differences therefore might be considered random too. Since pineal cysts were found to be very symmetric all of the time, we do not expect the difference found to be due to unilateral compression of draining veins.

## Conclusion

Diagnostic tools in a clinical environment must be easily reproducable and provide for clear and measurable differences. Eide and Ringstad delivered promising evidence on the pathogenesis of symptoms in Pineal cyst patients but ADC ratios do not seem to provide for a proper diagnostic tool to determine diagnosis and indication for surgical resection. Our data—and also that of Eide and Ringstad—suggests that probable changes are minor and sometimes contradicting. Analyses rather showed that variability is high and results hard to reproduce. Even though we did not find much evidence we still think that the hypothesis cannot be discarded and more research has to be done.

In following researches on this topic we suggest the protocol of Eide and Ringstad to be followed at least in data extraction. If possible, analysis could also be done comparing raw values when MRI was done using a field strength of 1.5 Tesla for standardization and avoiding bias. Given this we would not suggest to form ADC ratios in reference to central white matter.

## Data Availability

Data is available upon direct request addressed to stephan.greisert@med.uni-greifswald.de and after approval of the Ethical Review Board.

## Abbreviations

ADC: Apparent Diffusion Coefficient
MRI: Magnetic Resonance Imaging
DWI: Diffusion Weighted Imaging
PC: Pineal cyst
CSF: Corticospinal fluid
TSCR: Tectum-splenium/cyst ratio
